# The Influence of Chitosan on the Chemical Composition of Wines Fermented with *Lachancea thermotolerans*

**DOI:** 10.3390/foods13070987

**Published:** 2024-03-23

**Authors:** Javier Vicente, Luka Vladic, Domingo Marquina, Silvia Brezina, Doris Rauhut, Santiago Benito

**Affiliations:** 1 Unit of Microbiology, Genetics, Physiology and Microbiology Department, Biology Faculty, Complutense University of Madrid, Ciudad Universitaria S/N, 28040 Madrid, Spain; javievic@ucm.es (J.V.); dommarq@bio.ucm.es (D.M.); 2 Department of Food Science and Technology, University of Natural Resources and Life Sciences, Gregor-Mendel-Straße 33, 1180 Vienna, Austria; lukavladic10@gmail.com; 3 Department of Microbiology and Biochemistry, Hochschule Geisenheim University (HGU), Von-Lade-Straße 1, 65366 Geisenheim, Germany; silvia.brezina@hs-gm.de (S.B.); doris.rauhut@hs-gm.de (D.R.); 4 Department of Chemistry and Food Technology, Polytechnic University of Madrid, Ciudad Universitaria S/N, 28040 Madrid, Spain

**Keywords:** *Lachancea thermotolerans*, chitosan, L-lactic acid, organic acids, aroma compounds, wine

## Abstract

Chitosan exerts a significant influence on various chemical parameters affecting the quality of wine produced using multiple strains of *Lachancea thermotolerans*. The impact of chitosan on these parameters varies depending on the specific strain studied. We observed that, under the influence of chitosan, the fermentation kinetics accelerated for all examined strains. The formation of lactic acid increased by 41% to 97% across the studied *L. thermotolerans* strains, depending on the specific strain. This effect also influenced acidity-related parameters such as total acidity, which increased by 28% to 60%, and pH, which experienced a decrease of over 0.5 units. The consumption of malic acid increased by 9% to 20% depending on the specific strain of *L. thermotolerans*. Nitrogen consumption also rose, as evidenced by all *L. thermotolerans* strains exhibiting a residual value of Primary Amino Nitrogen (PAN) of below the detection limit, and ammonia consumption increased by 90% to 100%, depending on the strain studied. However, certain parameters such as acetic acid, succinic acid, and glycerol showed contradictory results depending on the strain under investigation. In terms of volatile composition, chitosan supplementation led to increased production of i-butanol by 32% to 65%, 3-methylbutanol by 33% to 63%, and lactic acid ethyl ester by 58% to 91% across all studied strains of *L. thermotolerans*. Other analyzed aroma compounds exhibited varying changes depending on the specific strain of *L. thermotolerans*.

## 1. Introduction

The use of non-*Saccharomyces* yeast species in the field of enology has witnessed a significant surge in recent decades [1,2]. These yeast species, and in some cases, specific strains within them, exhibit distinct abilities that differ from the widely used *Saccharomyces cerevisiae* in winemaking. These abilities have been demonstrated to have a positive impact on various wine quality parameters, including aroma compounds, acidity, polysaccharide concentration, glycerol content, final ethanol levels, and food safety. Several non-*Saccharomyces* species have been extensively studied from a scientific perspective, including *Torulaspora delbrueckii*, *Metschnikowia pulcherrima*, *Hanseniaspora uvarum* [3], *Hanseniaspora vineae*, *Schizosaccharomyces pombe*, *Pichia kluyveri*, and *Lachancea thermotolerans* [4,5,6,7,8]. The growing interest in these yeast species has prompted yeast manufacturers to include them in their commercial product offerings [9]. Consequently, winemakers worldwide can now capitalize on the advantages presented by non-*Saccharomyces* yeasts in their winemaking practices.

*L. thermotolerans* stands out among non-*Saccharomyces* and *Saccharomyces* yeasts as the only yeast capable of effectively acidifying wine during alcoholic fermentation [10]. In addition to its acidification ability, it offers other secondary advantages, such as reducing volatile acidity [4]; consuming malic acid [11,12]; decreasing final ethanol concentration [13]; and increasing the concentration of desirable volatile compounds like ethyl phenyl acetate [14]. However, there are important limitations to consider, including its moderate fermentative power [15] and limited resistance to sulfur dioxide [13]. To address the issue of reduced fermentative power, *L. thermotolerans* can be combined with more fermentative yeast species such as *S. cerevisiae* or *S. pombe* [8]. The limited sulfur dioxide resistance of *L. thermotolerans* can be addressed by using alternative compounds instead of sulfur dioxide. Some of these alternatives can inhibit spoilage microorganisms such as bacteria or *Brettanomyces/Dekkera* and prevent oxidation while allowing *L. thermotolerans* to carry out fermentation. Chitosan emerges as a promising option to combine with *L. thermotolerans* and other non-*Saccharomyces* yeasts unaffected by this antimicrobial agent.

Chitosan, a natural polysaccharide polymer, is gaining popularity in the winemaking industry [16,17,18] due to its antioxidant [19,20] and antimicrobial properties [21,22], which are particularly relevant given the ongoing trend towards reducing sulfur dioxide levels in wines [23]. Commercial chitosan-containing products, although more costly, serve as microbial control agents against bacteria such as lactic and acetic acid bacteria [22,24,25,26], as well as spoilage yeasts like *Brettanomyces* [27,28,29,30]. It also prevents browning effects in white wine and acts as a metal chelator, facilitating the control of heavy metals like iron and copper [31]. Additionally, chitosan aids clarification [32,33], promotes protein stability, and reduces contaminants like ochratoxin A [14]. Notably, chitosan exhibits a comparatively lower antimicrobial effect than sulfur dioxide, and its solubility under must and wine conditions is limited. However, it offers the advantage of being non-allergenic, unlike chitosan derived from fungal sources. Despite its widespread use in food technology, the application of chitosan in the wine industry is a relatively recent development, leading to a scarcity of research exploring its effects on various oenological parameters beyond antimicrobial control. Nonetheless, commercial chitosan products are readily available in most wine regions.

A previous study investigated the effects of chitosan on non-*Saccharomyces* yeasts, specifically *S. pombe* [34]. The study examined various parameters of the final wine composition, including acetic acid, ethanol, glycerol, acetaldehyde, pyruvic acid, α-ketoglutarate, higher alcohols, acetate esters, ethyl esters, and fatty acids. The results revealed that chitosan exerted an influence on these parameters. For one strain of *S. cerevisiae*, slight increases were observed in acetate esters, ethyl esters, and higher alcohols [34]. In contrast, the selected strain of *S. pombe* showed a significant reduction in the final concentrations of acetate esters, ethyl esters, and higher alcohols [34]. The effect of chitosan on fatty acids and terpenes was found to be less pronounced [34].

This study examines how chitosan affects the fermentation process of *L. thermotolerans*, as well as its impact on the aroma of the final wine, marking the first investigation of its kind.

## 2. Materials and Methods

### 2.1. Microorganisms

This study used the following *L. thermotolerans* yeast strains: NG-108, A11-612, EM-119, MJ-311, BD-612, L1, and L3 (Complutense University of Madrid, Madrid, Spain); Concerto (CHR Hansen, Hørsholm, Denmark); Laktia (Lallemand, Montréal, QC, Canada); Levulia Alcomeno (AEB Group, Brescia, Italy); EnartisFerm QK (Enartis, San Martino, Italy); Excellence (X’Fresh Lamothe-Abiet, Bordeaux, France); and Octave (CHR Hansen, Denmark). As a control, the *S. cerevisiae* strain AWRI-796 (Maurivin, Minto, Australia) was included. The identities of all yeast strains used in this research have been previously confirmed as unique strains through the application of the microsatellite genotyping protocol specifically designed for *L. thermotolerans* [35].

### 2.2. Vinification

Fermentations were conducted in triplicate using 100 mL borosilicate bottles, each containing 90 mL of Synthetic Grape Must (SGM), and maintained at a temperature of 25 °C. The SGM was prepared based on its original formulation, with slight modifications [35]. In brief, equimolar concentrations of glucose and fructose at 200 g/L, 3 g/L of malic acid, and 2.5 g/L of potassium tartrate were added to the SGM. The pH was adjusted to 3.5, and the nitrogen content was adjusted to 140 mg/L from amino acids and 60 mg/L from di-ammonium phosphate, according to the original formulation. Prior to fermentation, yeast precultures were incubated in SGM for 24 h at 25 °C under orbital shaking at 150 rpm. For inoculation, the final concentration of the yeast cells was adjusted to 2 × 10^5^ cells/mL (≈O.D λ_600nm_ = 0.02). Fermentation progress was monitored by measuring weight loss every 24 h. After the fermentations slowed down to a weight loss of less than 0.01% per day, the cultures were centrifuged at 7000 rpm for 5 min and then preserved at 4 °C for further analysis. The experiment’s design essentially consisted of a regular control fermentation using Synthetic Grape Must (SGM) for the studied yeast strains, and an additional trial enriched with 0.5 g/L of the commercial product BactilessTM (Lallemand, Canada). BactilessTM contains chitosan of fungal origin, which allows us to study the influence of this compound during the fermentations. BactilessTM is a 100% natural, non-GMO, and non-allergenic biopolymer derived from the fungus *Aspergillus niger*. Originally utilized to control bacterial populations in wines, BactilessTM is reported by the manufacturer to be effective against a wide spectrum of bacteria while not affecting yeast populations.

### 2.3. Chemical Parameter Measurements

The quantification of L-malic acid, L-lactic acid, ammonia, and Primary Amino Nitrogen (PAN) was performed using a Y15 Autoanalyzer along with commercially available kits from Biosystems (Barcelona, Spain) [36]. The determination of acetic acid, ethanol, glucose + fructose, succinic acid, total acidity, pH, and glycerol concentrations was conducted using the FTIR autoanalyzer Bacchus 3 (TDI, Barcelona, Spain) [37].

### 2.4. Volatile Compounds

The analysis of esters, higher alcohols, and fatty acids was conducted using the method developed by the Department of Microbiology and Biochemistry at Hochschule Geisenheim University, as previously reported in relevant studies [38].

### 2.5. Statistical Analyses

All statistical analyses were performed using R software version 4.1.2 (R Developement Core Team, Vienna, Austria, 2013). The significance level was set at *p* < 0.05. Analysis of variance (ANOVA) and Tukey post-hoc tests were applied to compare the different groups and values.

## 3. Results and Discussion

### 3.1. Fermentation Kinetics

**Figure 1:**
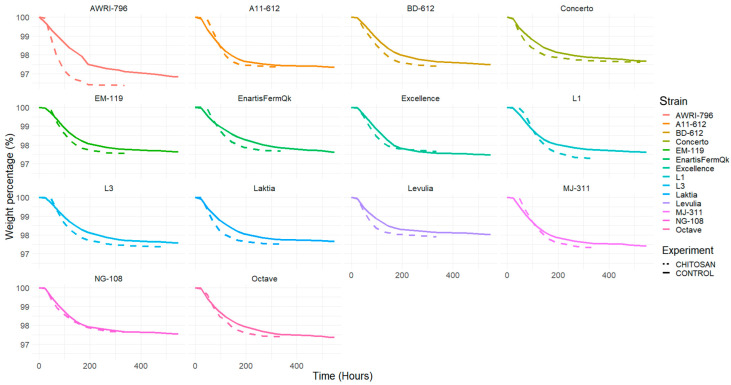
Fermentation kinetics of variants, gravimetrically measured by total weight loss during the pure fermentation of SGM examinates, for all the studied strains. Solid lines depict the pure fermentation of regular SGM, while dashed lines stand for the pure fermentation of SGM enriched with chitosan.

The control groups without chitosan required approximately 500 to 600 h for all studied strains of *L. thermotolerans* to reach a stationary phase, in which weight loss every 24 h was lower than 0.01%. However, the chitosan-treated trials, for the most part, reached this stage between 300 and 350 h, except for the *L. thermotolerans* strains Concerto and L3, which required around 500 h (Figure 1). In contrast, the *S. cerevisiae* control group took 1000 h to complete fermentation, whereas the chitosan-treated *S. cerevisiae* group reached the stationary stage in 350 h. These findings suggest that chitosan consistently accelerated the fermentation kinetics in all cases. This effect could be beneficial in mitigating the risk of sluggish alcoholic fermentation, although it should be noted that faster kinetics may require additional cooling measures at an industrial scale.

### 3.2. Glucose and Fructose

**Figure 2:**
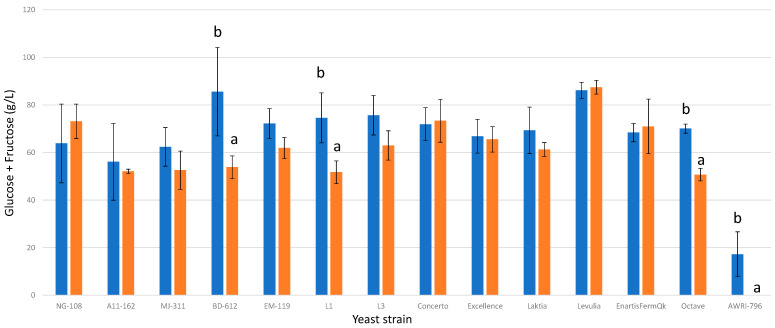
The final glucose + fructose concentrations (g/L) of the final wines fermented by the examined yeast strains are illustrated, indicating their fermentation in chitosan-free SGM (blue) and chitosan-enriched SGM (orange). Distinct letters are used to indicate statistically significant differences at a significance level of *p* = 0.05.

All trials involving *L. thermotolerans* strains exhibited high final sugar concentrations exceeding 40 g/L (Figure 2). These findings align with previous studies that recommend utilizing *L. thermotolerans* in conjunction with more fermentative yeast genera, such as *Saccharomyces* or *Schizosaccharomyces* [8], in mixed or sequential fermentations to ensure the complete consumption of residual sugars when the primary objective is to produce dry wine [8]. Although most *L. thermotolerans* strains displayed increased consumption of glucose and fructose under the influence of chitosan, only three strains exhibited statistically significant differences. Specifically, in the chitosan-enriched trials, the *L. thermotolerans* strains BD-612, L3, and Octave demonstrated enhanced glucose and fructose consumption by 37%, 30%, and 27%, respectively. In comparison, the *S. cerevisiae* strain consumed all sugars in the chitosan-treated trial, while the regular control, without chitosan, attained a final glucose and fructose concentration of 17 g/L.

### 3.3. Ethanol

**Figure 3:**
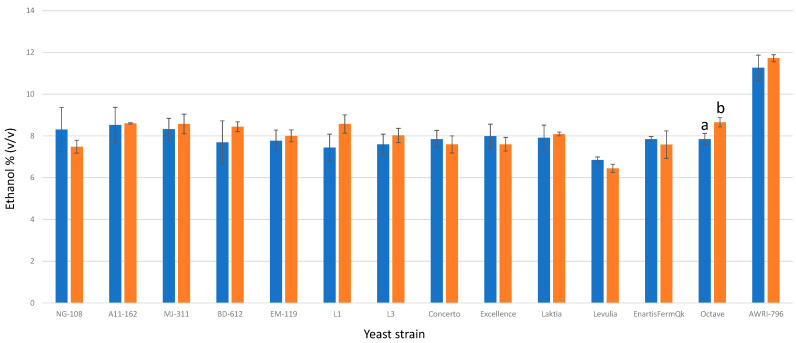
The final ethanol concentration percentages (*v*/*v*) of the final wines fermented by the examined yeast strains are illustrated, indicating their fermentation in chitosan-free SGM (blue) and chitosan-enriched SGM (orange). Distinct letters are used to indicate statistically significant differences at a significance level of *p* = 0.05.

The final ethanol concentrations varied from 6.45% to 8.65% (*v*/*v*) for the investigated *L. thermotolerans* strains (Figure 3). These results are consistent with prior studies, suggesting that *L. thermotolerans* species have the ability to ferment between 5% and 10% (*v*/*v*) in pure alcoholic fermentations [8]. While some trials exhibited slight increases in ethanol content for specific *L. thermotolerans* strains, only one strain out of the thirteen we examined demonstrated statistically significant differences. The commercial strain Octave displayed an ethanol production rate that was 0.8% (*v*/*v*) higher under chitosan conditions compared to the regular control without chitosan. Chitosan did not have an influence on the ethanol content of the *S. cerevisiae* control. This observation aligns with a previous study that reported no impact on the ethanol production rates of *S. cerevisiae* or *S. pombe* [34].

### 3.4. l-Lactic Acid

**Figure 4:**
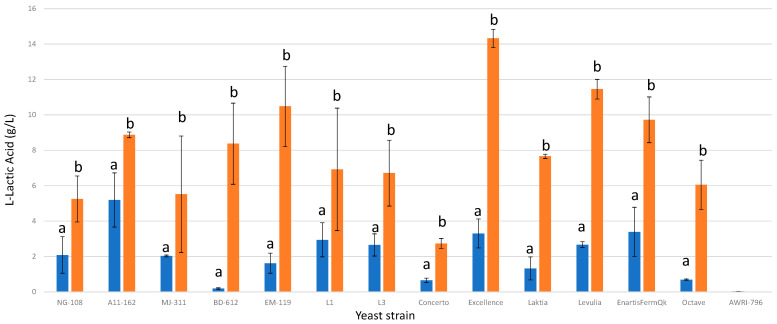
The final L-lactic acid concentrations (g/L) of the final wines fermented by the examined yeast strains are illustrated, indicating their fermentation in chitosan-free SGM (blue) and chitosan-enriched SGM (orange). Distinct letters are used to indicate statistically significant differences at a significance level of *p* = 0.05.

The addition of chitosan significantly enhanced lactic acid production in all examined strains of *L. thermotolerans* (Figure 4). The extent of the increase varied significantly, ranging from 41% to 97%, indicating the occurrence of different reactions depending on the specific *L. thermotolerans* strain. Among the strains, the commercial strain Excellence exhibited the most pronounced response. The final lactic acid concentrations ranged from 0.19 g/L to 5.19 g/L for the control groups without chitosan, whereas for the fermentations enriched with chitosan, the final values ranged from 2.74 g/L to 14.32 g/L. These findings suggest that, while the initial intention of employing chitosan in the management of *L. thermotolerans* was to address its reported sensitivity to sulfur dioxide [8], its utilization represents an intriguing option for enhancing the distinct capability of *L. thermotolerans* to produce lactic acid.

The potential impact of chitosan on the enhancement of lactic acid production can be multifaceted. Firstly, this polymer, characterized by its positively charged groups, possesses the capability to engage in electrostatic interactions with anions derived from dissociated organic acids [39]. Furthermore, the polymer may exert an influence on the permeability of the cellular membrane. Acting as a membrane-binding molecule, its interaction with the cellular membrane has the potential to impede the diffusion rates of weak organic acids towards the intracellular medium [40]. Consequently, this hindrance in diffusion may attenuate the consequential effects of these acids on cellular homeostasis, thereby facilitating heightened production.

### 3.5. Titratable Acidity

**Figure 5:**
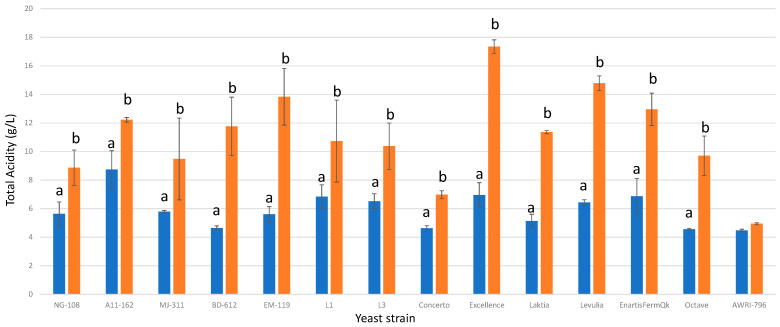
The final total acidity concentrations (g/L) of the final wines fermented by the examined yeast strains are illustrated, indicating their fermentation in chitosan-free SGM (blue) and chitosan-enriched SGM (orange). Distinct letters are used to indicate statistically significant differences at a significance level of *p* = 0.05.

The production of lactic acid caused a significant increase in total acidity across all studied strains of *L. thermotolerans*. The magnitude of this increase varied from 28% (strain A11-612) to 60% (strains BD-612 and Excellence), depending on the specific strain (Figure 5). In trials involving chitosan, the final concentrations of total acidity ranged from 6.97 g/L to 17.34 g/L, while in the regular control groups, they varied from 4.56 g/L to 8.74 g/L. Although the acidification effect was significant, certain values could be excessive and may potentially impede the performance of other yeast partners, such as *S. cerevisiae*. These results indicate that the influence of chitosan on total acidity must be assessed during selection processes to prevent excessive acidification or compatibility issues with *S. cerevisiae*. Previous studies have reported increases in total acidity of up to 10.4 g/L in *L. thermotolerans* strains without the influence of chitosan [8].

### 3.6. pH Values

**Figure 6:**
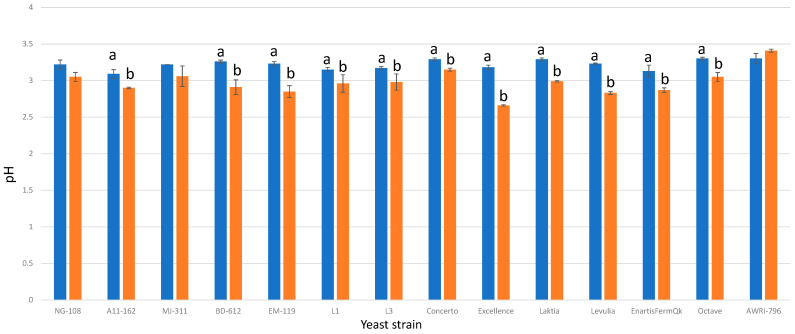
The final pH values of the final wines fermented by the examined yeast strains are illustrated, indicating their fermentation in chitosan-free SGM (blue) and chitosan-enriched SGM (orange). Distinct letters are used to indicate statistically significant differences at a significance level of *p* = 0.05.

In the fermentations enriched with chitosan, eleven out of thirteen strains of *L. thermotolerans* exhibited significant decreases in pH (Figure 6). Only two strains (NG-108 and MJ-311) did not demonstrate statistically significant differences. The regular control groups without chitosan displayed pH values ranging from 3.09 to 3.3, and were obtained from an SGM with an initial pH of 3.5. This reduction in pH can be attributed to lactic acid formation. Prior studies have reported pH decreases of up to 0.5 units; this closely aligns with the 0.41 unit decrease observed in strain A11-612 [8]. Conversely, the fermentations enriched with chitosan exhibited final pH values ranging from 2.66 to 3.15, representing decreases in pH ranging from 0.35 to 0.84 units. Nine strains displayed pH decreases exceeding 0.5 units (EnartirFermQK, Levulia, Laktia, Excellence, L3, L1, EM-119, BD-612, and A11-612). While pH reduction can be advantageous in certain scenarios, it is important to consider that excessive pH reductions could potentially compromise the alcoholic fermentation process or the performance of associated strains from other species, such as *S. cerevisiae*, which are essential for completing the alcoholic fermentation process under industrial conditions.

### 3.7. Malic Acid

**Figure 7:**
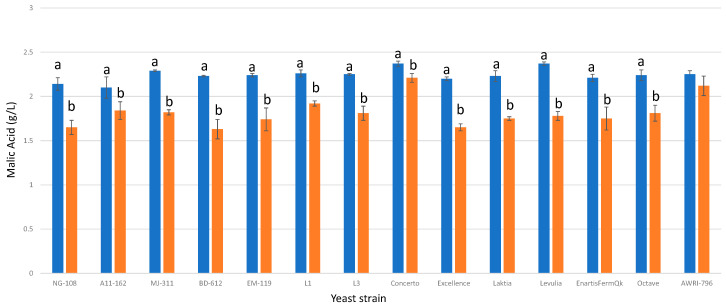
The final L-malic acid concentrations (g/L) of the final wines fermented by the examined yeast strains are illustrated, indicating their fermentation in chitosan-free SGM (blue) and chitosan-enriched SGM (orange). Distinct letters are used to indicate statistically significant differences at a significance level of *p* = 0.05.

All the studied *L. thermotolerans* strains demonstrated reductions in malic acid ranging from 20% to 30% (Figure 7). In all cases, the addition of chitosan intensified the effect of malic acid reduction, resulting in final values ranging from 25% to 45%. The increase in malic acid consumption varied from 9% for the A11-612 strain to 20% for the BD-612 strain. Recent studies highlight the consumption of malic acid as an important secondary selective parameter for *L. thermotolerans*, following the production of lactic acid [19,20]. This property is particularly desirable when producing red wines, as it is important to minimize the presence of malic acid prior to bottling to avoid unwanted refermentation. While no study has reported any *L. thermotolerans* strain capable of completely consuming all malic acid in red wine, specific *L. thermotolerans* strains have been shown to synergize with other oenological microorganisms, such as *Oenococcus oeni*, *Lactiplantibacillus plantarum*, or *S. pombe*, which are capable of consuming malic acid [10]. These results demonstrate that incorporating chitosan can be a compelling approach towards enhancing the desired reduction of malic acid during the production of red wine.

### 3.8. Acetic Acid

**Figure 8:**
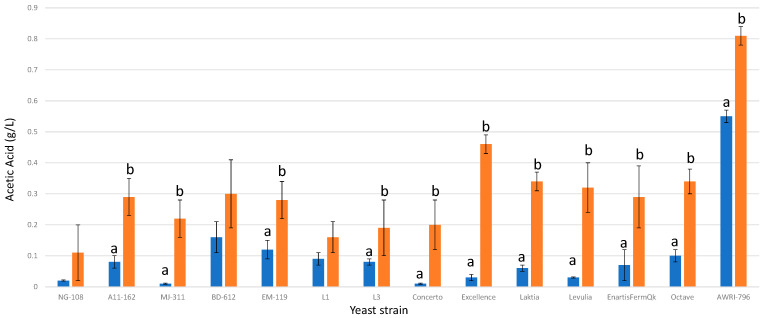
The final acetic acid concentrations (g/L) of the final wines fermented by the examined yeast strains are illustrated, indicating their fermentation in chitosan-free SGM (blue) and chitosan-enriched SGM (orange). Distinct letters are used to indicate statistically significant differences at a significance level of *p* = 0.05.

Chitosan supplementation led to increased production of acetic acid in 9 out of the 13 strains studied (Figure 8). However, all final concentrations remained well below the detectable threshold of 0.6–0.9 g/L [41] that can generally be associated with faulty vinegar characteristics, although it depends on the wine style. The strains NG-108, BD-612, EM-119, and L1 did not exhibit significant differences between the controls without chitosan and their chitosan-treated counterparts. Among the *L. thermotolerans* strains, the increases in acetic acid varied from 0.1 g/L (strain L3) to 0.4 g/L (strain Excellence). The final concentrations of acetic acid in the fermentations enriched with chitosan ranged from 0.11 g/L to 0.34 g/L, except for strain Excellence, which reached a significantly higher value of 0.46 g/L. Notably, strain Excellence had one of the lowest concentrations in the control group without chitosan. These results highlight the strain-dependent influence of chitosan on acetic acid production, emphasizing the need to consider this factor during the selection process of *L. thermotolerans* strains. The final concentrations of acetic acid in the control groups without chitosan were very low, below 0.1 g/L, which aligns with previous studies describing *L. thermotolerans* strains as lower producers of volatile acidity compared to *S. cerevisiae* [8]. A prior investigation, centered on the impact of chitosan on the non-*Saccharomcyes S. pombe*, revealed a noteworthy 0.1 g/L increase in acetic acid under the influence of chitosan [34].

### 3.9. Succinic Acid

**Figure 9:**
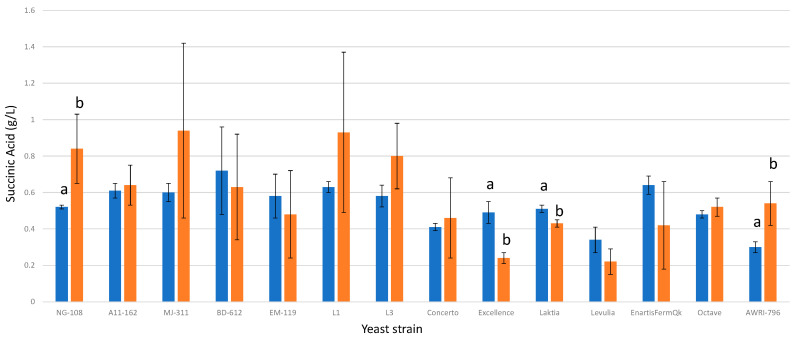
The final succinic acid concentrations (g/L) of the final wines fermented by the examined yeast strains are illustrated, indicating their fermentation in chitosan-free SGM (blue) and chitosan-enriched SGM (orange). Distinct letters are used to indicate statistically significant differences at a significance level of *p* = 0.05.

The importance of yeast strain selection has gained prominence in recent years due to the association of succinic acid with the sensory descriptor of minerality, a distinctive parameter in certain wines [10]. *S. cerevisiae* exhibits strain variability in succinic acid production, ranging from 0.5 g/L to 1.8 g/L (Figure 9). The average values of succinic acid concentrations in the regular control groups without chitosan varied from 0.34 g/L to 0.72 g/L, while in the fermentations enriched with chitosan, the values ranged from 0.22 g/L to 0.94 g/L for the studied *L. thermotolerans* strains. However, only three out of the thirteen *L. thermotolerans* strains displayed statistically significant differences. One strain demonstrated a higher final concentration of succinic acid (strain NG-108), with an increase of 0.32 g/L compared to the regular control without chitosan, while two strains exhibited reduced final concentrations (strains Excellence and Laktia), with decreases of 0.25 g/L and 0.08 g/L, respectively. Under the influence of chitosan, the *S. cerevisiae* control group exhibited an increase of 0.24 g/L in the final concentration of succinic acid. These findings suggest that only a limited number of strains demonstrate a notable impact on succinic acid production under the influence of chitosan.

### 3.10. Glycerol

**Figure 10:**
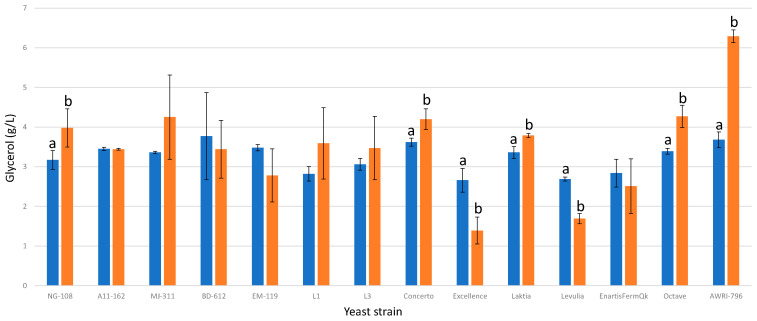
The final glycerol concentrations (g/L) of the final wines fermented by the examined yeast strains are illustrated, indicating their fermentation in chitosan-free SGM (blue) and chitosan-enriched SGM (orange). Distinct letters are used to indicate statistically significant differences at a significance level of *p* = 0.05.

Chitosan influenced the glycerol production of six out of the thirteen studied strains of *L. thermotolerans* (Figure 10). Among these, four strains (NG-108, Concerto, Laktia, and Octave) exhibited moderate but statistically significant increases in their final concentrations of glycerol, of 20%, 13%, 11%, and 20%, respectively. On the other hand, two strains (Excellence and Levulia) displayed the opposite effect, significantly reducing their final glycerol concentrations by 47% and 37%, respectively. Previous studies have highlighted substantial variability in glycerol production attributed to the strain of *L. thermotolerans* [8]. Similar effects have been observed in other yeast genera, such as *Saccharomyces* and *Schizosaccharomyces* [34].

### 3.11. Ammonia

**Figure 11:**
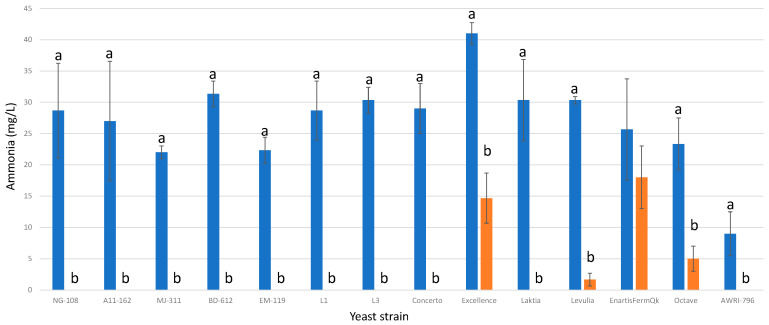
The final ammonia concentrations (mg/L) of the final wines fermented by the examined yeast strains are illustrated, indicating their fermentation in chitosan-free SGM (blue) and chitosan-enriched SGM (orange). Distinct letters are used to indicate statistically significant differences at a significance level of *p* = 0.05.

Chitosan exerted a significant influence on ammonia consumption for twelve out of the thirteen studied strains of *L. thermotolerans* (Figure 11). The control groups without chitosan displayed final ammonia concentrations ranging from 22 mg/L to 41 mg/L, while fermentations enriched with chitosan predominantly resulted in final concentrations of 0 mg/L for most strains (NG-108, A11-612, MJ-311, BD-612, EM-119, L1, L3, Concerto, and Laktia). Strain Excellence exhibited a final ammonia concentration 64% lower than the control without chitosan, while Levulia and Concerto showed reductions of 95% and 79%, respectively. The only *L. thermotolerans* strain that did not display significant differences between the control and the chitosan-treated version was strain EnartisFermQK. This finding highlights the importance of considering nutrient deficiencies when utilizing chitosan, as it may lead to undesired situations of nutrient shortage, potentially affecting the performance of more fermentative yeasts, like *S. cerevisiae*, which typically concludes the alcoholic fermentation process in sequential combinations with *L. thermotolerans* at an industrial scale. To mitigate these potential issues, a second addition of nutrients during the alcoholic fermentation process could be implemented [8].

### 3.12. Primary Amino Nitrogen

**Figure 12:**
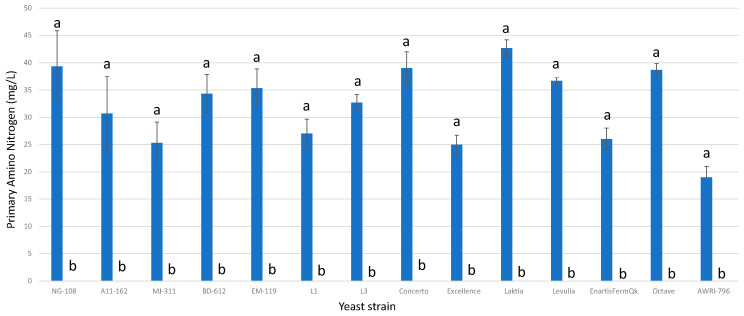
The final PAN concentrations (mg/L) of the final wines fermented by the examined yeast strains are illustrated, indicating their fermentation in chitosan-free SGM (blue) and chitosan-enriched SGM (orange). Distinct letters are used to indicate statistically significant differences at a significance level of *p* = 0.05.

All fermentations enriched with chitosan exhibited final concentrations of PAN of 0 mg/L (Figure 12), while the regular control groups without chitosan displayed values ranging from 25 mg/L to 42 mg/L. Despite the significant advantages observed, such as increased acidity and lactic acid production, the higher demand for nutrients such as ammonia and PAN indicates the need for optimized management to avoid potential technical issues at an industrial scale. This effect may also be of interest during the production of wines with low levels of biogenic amines, as the absence of amino acids would reduce the precursors of undesirable hazardous compounds, such as biogenic amines.

### 3.13. Volatile Compounds

So far, there have not been any studies exploring how chitosan impacts the aroma of wine, particularly when fermented by *L. thermotolerans*. This lack of research makes direct comparisons impossible. However, a previous study examined the effects of chitosan on another non-*Saccharomyces* yeast species, *S. pombe* [34]. That study reported that a specific strain of *S. pombe* produced lower levels of 3-methylbutanol, 2-phenylethanol, acetic acid ethyl ester, acetic acid 3-methylbutyl ester, acetic acid 2-methylbutyl ester, acetic acid hexyl ester, acetic acid 2-phenylethyl ester, butyric acid ethyl ester, hexanoic acid ethyl ester, decanoic acid ethyl ester, isovaleric acid, hexanoic acid, and decanoic acid. Conversely, increases in i-butyric acid ethyl ester and propionic acid ethyl ester were observed. While this study provides insights into the potential impact of chitosan on aroma composition in non-*Saccharomyces* yeast fermentations, further research is necessary to understand its specific effects on *L. thermotolerans* strains.

#### 3.13.1. i-Butanol

**Figure 13:**
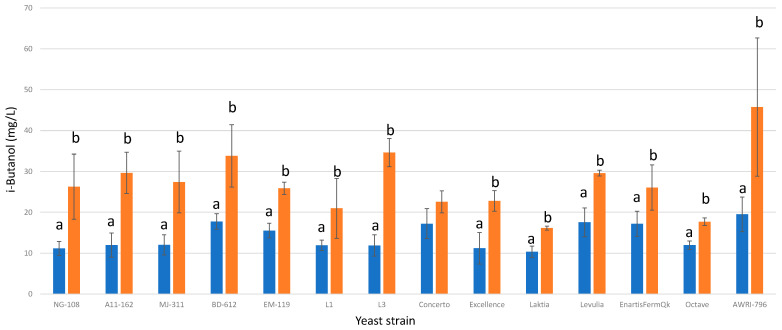
The final i-butanol concentrations (mg/L) of the final wines fermented by the examined yeast strains are illustrated, indicating their fermentation in chitosan-free SGM (blue) and chitosan-enriched SGM (orange). Distinct letters are used to indicate statistically significant differences at a significance level of *p* = 0.05.

Under the influence of chitosan, twelve out of the thirteen strains of *L. thermotolerans* exhibited a significant increase in i-butanol production. Strains NG-108, A11-612, MJ-311, BD-612, EM-119, L1, L3, Excellence, Laktia, Levulia, EnartisFermQK, and Octave demonstrated increases of 57%, 59%, 56%, 47%, 39%, 43%, 65%, 50%, 36%, 40%, 34%, and 32%, respectively (Figure 13). In comparison, the *S. cerevisiae* control group displayed a 57% higher i-butanol production rate in the presence of chitosan compared to the regular control without chitosan. These findings emphasize the potential of chitosan in enhancing i-butanol production in *L. thermotolerans* strains, as well as its impact on *S. cerevisiae*. In a single prior study detailing the impact of chitosan on a non-*Saccharomyces* during fermentation [34], it is noted that a specific strain of *Schizosaccharomyces pombe* exhibited a 15% higher concentration of i-butanol compared to the *S. pombe* control undergoing alcoholic fermentation without chitosan influence.

#### 3.13.2. 3-Methylbutanol

**Figure 14:**
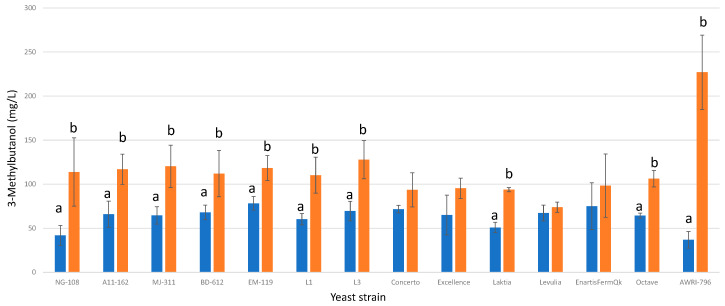
The final 3-methylbutanol concentrations (mg/L) of the final wines fermented by the examined yeast strains are illustrated, indicating their fermentation in chitosan-free SGM (blue) and chitosan-enriched SGM (orange). Distinct letters are used to indicate statistically significant differences at a significance level of *p* = 0.05.

All the studied strains of *L. thermotolerans* exhibited higher final concentrations of 3-methylbutanol when fermented in chitosan-enriched synthetic must (Figure 14). Among the thirteen strains, nine demonstrated statistically significant differences. Specifically, strains NG-108, A11-612, MJ-311, BD-612, EM-119, L1, L3, Laktia, and Octave displayed final concentrations of 3-methylbutanol that were increased by 63%, 43%, 46%, 39%, 33%, 45%, 45%, 45%, and 39%, respectively, under the influence of chitosan. These findings highlight the significant impact of chitosan on enhancing 3-methylbutanol production during *L. thermotolerans* fermentation. Within the confines of the lone preceding study exploring the effects of chitosan on a non-*Saccharomyces* during fermentation [34], it is highlighted that a particular strain of *Schizosaccharomyces pombe* yielded a 25% lower concentration of 3-methylbutanol than the *S. pombe* control undergoing alcoholic fermentation without chitosan influence.

#### 3.13.3. 2-Methylbutanol

**Figure 15:**
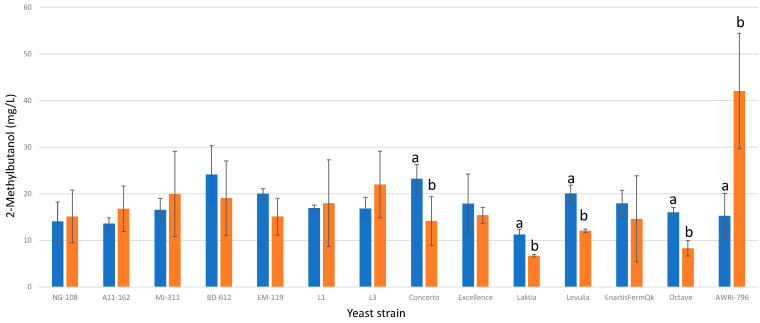
The final 2-methylbutanol concentrations (mg/L) of the final wines fermented by the examined yeast strains are illustrated, indicating their fermentation in chitosan-free SGM (blue) and chitosan-enriched SGM (orange). Distinct letters are used to indicate statistically significant differences at a significance level of *p* = 0.05.

Out of the studied strains of *L. thermotolerans*, only four exhibited statistical differences in 2-methylbutanol production. Specifically, strains Concerto, Laktia, Levulia, and Octave demonstrated significantly lower levels of 2-methylbutanol, with reductions of 39%, 40%, 39%, and 48%, respectively, in their chitosan-enriched fermentations compared to the regular controls (Figure 15). In contrast, the *S. cerevisiae* control group showed a 63% increase in 2-methylbutanol production in their chitosan-enriched fermentations. These findings emphasize the strain-specific effects of chitosan on 2-methylbutanol production in *L. thermotolerans* and its contrasting impact on *S. cerevisiae*. The sole prior study examining the influence of chitosan on a non-*Saccharomyces* during fermentation [34] reports that a selected strain of *Schizosaccharomyces pombe* produced an 11% higher concentration of 2-methylbutanol than the *S. pombe* control undergoing alcoholic fermentation without chitosan influence.

#### 3.13.4. 2-Phenylethanol

**Figure 16:**
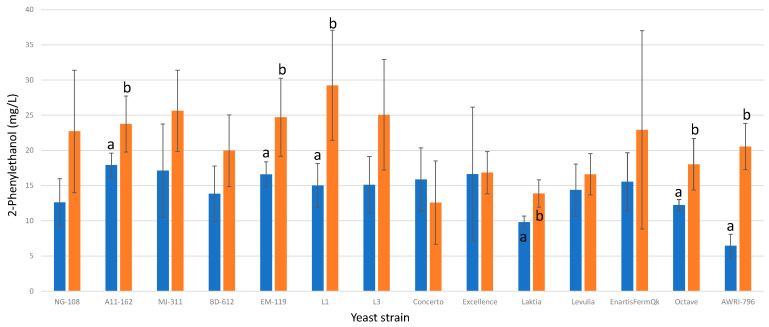
The final 2-phenylethanol concentrations (mg/L) of the final wines fermented by the examined yeast strains are illustrated, indicating their fermentation in chitosan-free SGM (blue) and chitosan-enriched SGM (orange). Distinct letters are used to indicate statistically significant differences at a significance level of *p* = 0.05.

Among the *L. thermotolerans* strains investigated, five strains (A11-612, EM-119, L1, Laktia, and Octave) exhibited significantly higher final concentrations of 2-phenylethanol (Figure 16). Under chitosan conditions, these strains displayed increases of 24%, 32%, 48%, 29%, and 32%, respectively. These findings highlight the ability of chitosan to enhance the production of 2-phenylethanol in select *L. thermotolerans* strains. In the only previous study on the influence of chitosan on a non-*Saccharomyces* during fermentation [34], it is highlighted that a specific strain of *Schizosaccharomyces pombe* presented a 16% lower concentration of 3-methylbutanol compared to the *S. pombe* control undergoing alcoholic fermentation without chitosan influence. Notably, unlike *L. thermotolerans*, no prior study characterizes *S. pombe* as a significant producer of 2-phenylethanol.

#### 3.13.5. Lactic Acid Ethyl Ester

**Figure 17:**
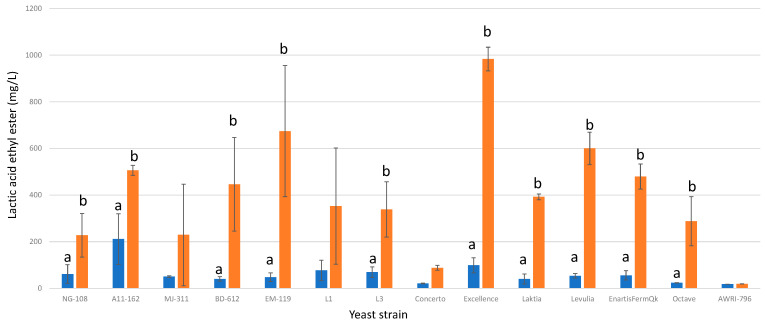
The final lactic acid ethyl ester concentrations (mg/L) of the final wines fermented by the examined yeast strains are illustrated, indicating their fermentation in chitosan-free SGM (blue) and chitosan-enriched SGM (orange). Distinct letters are used to indicate statistically significant differences at a significance level of *p* = 0.05.

All the studied strains of *L. thermotolerans* exhibited higher final concentrations of lactic acid ethyl ester when fermented in chitosan-enriched synthetic must (Figure 17). Among the thirteen strains, eleven demonstrated statistically significant differences. This effect can be attributed to the increased lactic acid production observed in the fermentations involving chitosan. Specifically, strains NG-108, A11-612, BD-612, EM-119, L3, Concerto, Excellence, Laktia, Levulia, EnartisFermQK, and Octave produced significantly higher levels of lactic acid ethyl ester, with increases of 72%, 58%, 90%, 92%, 79%, 76%, 89%, 89%, 91%, 88%, and 91%, respectively, when fermented with chitosan. These findings highlight the substantial impact of chitosan on enhancing lactic acid ethyl ester production during *L. thermotolerans* fermentation.

#### 3.13.6. Acetic Acid Ethyl Ester

**Figure 18:**
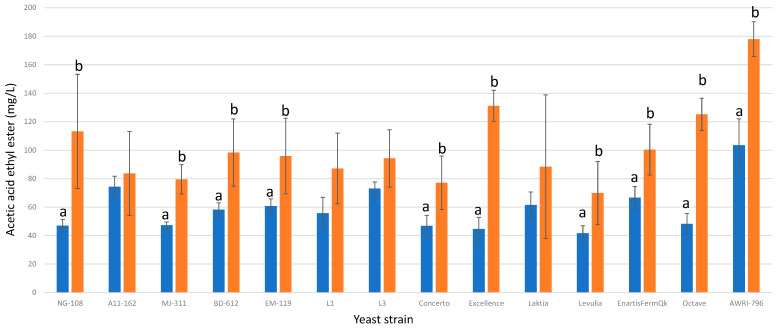
The final acetic acid ethyl ester concentrations (mg/L) of the final wines fermented by the examined yeast strains are illustrated, indicating their fermentation in chitosan-free SGM (blue) and chitosan-enriched SGM (orange). Distinct letters are used to indicate statistically significant differences at a significance level of *p* = 0.05.

The addition of chitosan to fermentations resulted in higher final concentrations of acetic acid ethyl ester in all cases. This effect can be attributed to the increased production of acetic acid observed under chitosan conditions, as mentioned previously. Among the thirteen studied strains of *L. thermotolerans*, nine exhibited statistically significant differences between the regular control and the chitosan-enriched fermentation (Figure 18). Specifically, strains NG-108, MJ-311, BD-612, EM-119, Concerto, Excellence, Levulia, EnartisFermQK, and Octave showed significantly higher levels of acetic acid ethyl ester, with increases of 58%, 40%, 40%, 36%, 39%, 66%, 40%, 33%, and 61%, respectively. In the *S. cerevisiae* control group with chitosan, there was a significant increase of 41% in acetic acid ethyl ester production. These findings demonstrate the pronounced impact of chitosan on enhancing acetic acid ethyl ester production in *L. thermotolerans* fermentations, as well as its effect on *S. cerevisiae*. In an earlier study, it was observed that chitosan led to a 28% decrease in ethyl acetate for *S. pombe*, while the control group with *S. cerevisiae* showed a 9% increase [34].

#### 3.13.7. Propionic Acid Ethyl Ester

**Figure 19:**
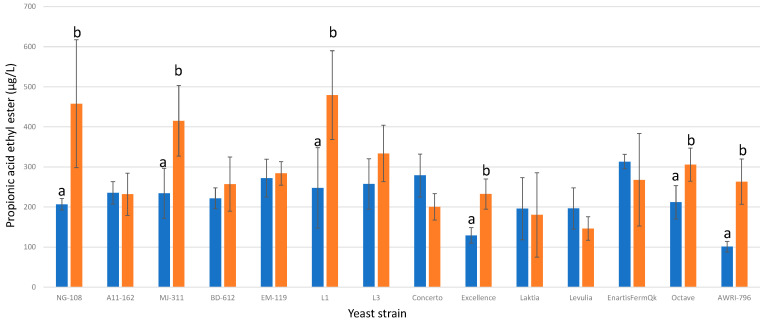
The final propionic acid ethyl ester concentrations (µg/L) of the final wines fermented by the examined yeast strains are illustrated, indicating their fermentation in chitosan-free SGM (blue) and chitosan-enriched SGM (orange). Distinct letters are used to indicate statistically significant differences at a significance level of *p* = 0.05.

Among the 13 studied strains of *L. thermotolerans*, only 5 demonstrated significant statistical differences between their regular controls and chitosan-enriched trials (Figure 19). These strains include NG-108, MJ-311, L1, Excellence, and Octave, all of which exhibited higher concentrations in their chitosan-enriched fermentations compared to the regular controls, with increases of 58%, 43%, 48%, 44%, and 30%, respectively. These findings highlight the strain-specific responses to chitosan and underscore the importance of considering individual strain characteristics when evaluating the effects of chitosan on *L. thermotolerans* fermentations. Examining the influence of chitosan on the non-*Saccharomyces S. pombe*, a previous study reported a 28% rise in propionic acid ethyl ester [34].

#### 3.13.8. i-Butyric Acid Ethyl Ester

**Figure 20:**
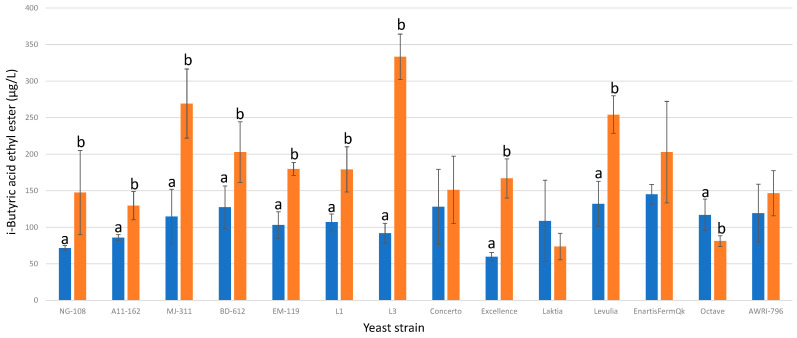
The final i-butyric acid ethyl ester concentrations (µg/L) of the final wines fermented by the examined yeast strains are illustrated, indicating their fermentation in chitosan-free SGM (blue) and chitosan-enriched SGM (orange). Distinct letters are used to indicate statistically significant differences at a significance level of *p* = 0.05.

When enriched with chitosan, nine strains of *L. thermotolerans* exhibited higher final concentrations of i-butyric acid ethyl ester compared to the regular controls, while one strain showed a decrease (Figure 20). Specifically, strains NG-108, A11-612, MJ-311, BD-612, EM-119, L1, L3, Excellence, and Levulia demonstrated increases of 51%, 33%, 57%, 37%, 42%, 40%, 72%, 64%, and 48%, respectively, in i-butyric acid ethyl ester production. In contrast, strain Octave showed a decrease of 31% in i-butyric acid ethyl ester production when enriched with chitosan. These findings highlight the strain-dependent effects of chitosan on i-Butyric acid ethyl ester production in *L. thermotolerans* fermentations. The sole preceding study investigating the impact of chitosan on a specific non-*Saccharomyces* strain, namely *S. pombe*, noted a 33% increase in i-butyric acid ethyl ester [34].

#### 3.13.9. Butyric Acid Ethyl Ester

**Figure 21:**
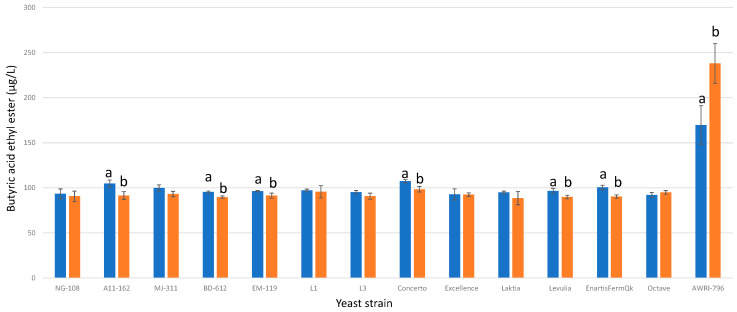
The final butyric acid ethyl ester concentrations (µg/L) of the final wines fermented by the examined yeast strains are illustrated, indicating their fermentation in chitosan-free SGM (blue) and chitosan-enriched SGM (orange). Distinct letters are used to indicate statistically significant differences at a significance level of *p* = 0.05.

Among the studied strains of *L. thermotolerans*, five strains exhibited slightly lower final concentrations of butyric acid ethyl ester when fermented with chitosan (Figure 21). Strains A11-612, BD-612, EM-119, Concerto, Levulia, and EnartisFermQK displayed decreases of 12%, 6%, 5%, 8%, 7%, and 10%, respectively, in butyric acid ethyl ester production during their chitosan-enriched fermentations compared to the regular controls. In contrast, the *S. cerevisae* strain showed an increase of 28% in butyric acid ethyl ester production for the trial involving chitosan compared to the regular control. These findings underscore the strain-specific effects of chitosan on butyric acid ethyl ester production in *L. thermotolerans* fermentations and highlight the contrasting impact on *S. cerevisiae*. An earlier investigation into the influence of chitosan on the non-*Saccharomyces S. pombe* documented a 42% decrease in butyric acid ethyl ester [34].

#### 3.13.10. Acetic Acid 3-Methylbutyl Ester

**Figure 22:**
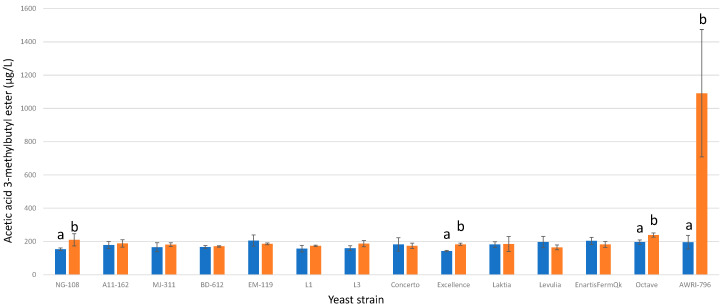
The final acetic acid 3-methylbutyl ester concentrations (µg/L) of the final wines fermented by the examined yeast strains are illustrated, indicating their fermentation in chitosan-free SGM (blue) and chitosan-enriched SGM (orange). Distinct letters are used to indicate statistically significant differences at a significance level of *p* = 0.05.

Among the studied strains of *L. thermotolerans*, only three strains demonstrated significant statistical differences in concentrations of acetic acid 3-methylbutyl ester (Figure 22). These strains—namely NG-108, Excellence, and Octave—exhibited increases of 26%, 22%, and 17%, respectively, when fermented with chitosan. These findings highlight the strain-specific responses to chitosan and underscore the importance of considering individual strain characteristics when evaluating its effects on *L. thermotolerans* fermentations. In previous research focusing on chitosan’s effects on non-*Saccharomyces* strains, specifically *S. pombe*, a 49% decrease in acetic acid 3-methylbutyl ester was reported under chitosan influence, while the *S. cerevisiae* control group exhibited a 29% increase [34].

#### 3.13.11. Acetic Acid 2-Methylbutyl Ester

**Figure 23:**
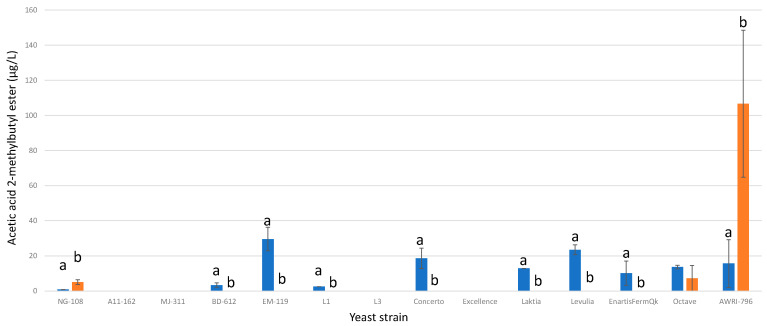
The final acetic acid 2-methylbutyl ester concentrations (µg/L) of the final wines fermented by the examined yeast strains are illustrated, indicating their fermentation in chitosan-free SGM (blue) and chitosan-enriched SGM (orange). Distinct letters are used to indicate statistically significant differences at a significance level of *p* = 0.05.

Seven strains of *L. thermotolerans* (BD-612, EM-119, L1, Concerto, Laktia, Levulia, and EnartisFermQK) exhibited no detectable production of acetic acid 2-methylbutyl ester when fermented with chitosan, while the controls yielded small amounts ranging from 2 to 30 µg/L (Figure 23). In contrast, the NG-108 strain displayed an opposing effect, producing 5.06 µg/L under chitosan influence compared to 0.89 µg/L for the regular controls. These findings highlight the strain-dependent variations in acetic acid 2-methylbutyl ester production during *L. thermotolerans* fermentation, emphasizing the impact of chitosan on this particular compound. The preceding research that addressed non-*Saccharomyces* and chitosan reported a 28% reduction in acetic acid 2-methylbutyl ester for *S. pombe* during fermentation with chitosan [34].

## 4. Conclusions

Chitosan did not exhibit a significant impact on the fermentative power of *L. thermotolerans*, but it did significantly affect several other kinetic and chemical parameters of oenological relevance. Notably, chitosan demonstrated a significant influence in increasing various acidification-related parameters, including lactic acid production, total acidity, and pH reduction for all the studied strains of *L. thermotolerans*. Therefore, chitosan represents an intriguing tool for enhancing the acidification potential of *L. thermotolerans*. Additionally, chitosan significantly influenced other oenological parameters such as malic acid consumption, PAN, i-butanol, 3-methylbutanol, and lactic acid ethyl ester. The impact of chitosan exhibited considerable strain variability, dependent on the specific *L. thermotolerans* strain under investigation. These findings highlight the multifaceted influence of chitosan on various oenological parameters, emphasizing the importance of strain selection when employing chitosan in *L. thermotolerans* fermentations.

## Data Availability

The original contributions presented in the study are included in the article, further inquiries can be directed to the corresponding author.
